# Getting More Power from Your Flowers: Multi-Functional Flower Strips Enhance Pollinators and Pest Control Agents in Apple Orchards

**DOI:** 10.3390/insects8030101

**Published:** 2017-09-20

**Authors:** Alistair John Campbell, Andrew Wilby, Peter Sutton, Felix Wäckers

**Affiliations:** 1 Embrapa Amazônia Oriental, Tv. Dr Enéas Pinheiro, S/N, Belém, PA 66095-100, Brazil; 2 Lancaster Environment Centre, Lancaster University, Lancaster LA1 4YQ, UK; a.wilby@lancaster.ac.uk; 3 Jealotts Hill International Research Centre, Syngenta UK, Jealott’s Hill, Bracknell RG42 6EY, UK; peter.sutton@syngenta.com; 4 Biobest Belgium NV, Ilse Velden 18, 2260 Westerlo, Belgium; felix.wackers@biobest.be

**Keywords:** agroecology, ecological intensification, agri-environment schemes, floral traits, conservation biological control, ecosystem services, beneficial arthropods

## Abstract

Flower strips are commonly recommended to boost biodiversity and multiple ecosystem services (e.g., pollination and pest control) on farmland. However, significant knowledge gaps remain regards the extent to which they deliver on these aims. Here, we tested the efficacy of flower strips that targeted different subsets of beneficial arthropods (pollinators and natural enemies) and their ecosystem services in cider apple orchards. Treatments included mixes that specifically targeted: (1) pollinators (‘concealed-nectar plants’); (2) natural enemies (‘open-nectar plants’); or (3) both groups concurrently (i.e., ‘multi-functional’ mix). Flower strips were established in alleyways of four orchards and compared to control alleyways (no flowers). Pollinator (e.g., bees) and natural enemy (e.g., parasitoid wasps, predatory flies and beetles) visitation to flower strips, alongside measures of pest control (aphid colony densities, sentinel prey predation), and fruit production, were monitored in orchards over two consecutive growing seasons. Targeted flower strips attracted either pollinators or natural enemies, whereas mixed flower strips attracted both groups in similar abundance to targeted mixes. Natural enemy densities on apple trees were higher in plots containing open-nectar plants compared to other treatments, but effects were stronger for non-aphidophagous taxa. Predation of sentinel prey was enhanced in all flowering plots compared to controls but pest aphid densities and fruit yield were unaffected by flower strips. We conclude that ‘multi-functional’ flower strips that contain flowering plant species with opposing floral traits can provide nectar and pollen for both pollinators and natural enemies, but further work is required to understand their potential for improving pest control services and yield in cider apple orchards.

## 1. Introduction

In the coming decades, agriculture must simultaneously meet the demands of feeding growing human populations while reducing its environmental impacts if we are to achieve goals for biodiversity conservation and food security [1]. Yield increases achieved using conventional farming practices (e.g., mechanisation, large field size, agrochemical usage) have come at a great cost to biodiversity [2,3,4], but also generate negative feedbacks for biodiversity-mediated ecosystem processes that underpin crop yields (e.g., pollination, pest control, nutrient cycling), thus potentially undermining agricultural production [5,6]. Consequently, there is growing interest in farming practices that harness the power of ecological functions for crop production (i.e., ‘ecological intensification’), and reduce our reliance on conventional inputs, that are increasingly costly and can have negative environmental impacts [7,8].

Arthropods provide many important ecosystem services on farmland, including the pollination of crops and control of damaging pest species [9,10]. Pollinators, but also many natural enemies (together termed ‘beneficial arthropods’) depend on flowering plants for essential nutrition (e.g., pollen and nectar) at some point in their life cycle. Consequently, loss and fragmentation of flower-rich habitats (e.g., forest edges, grassland, hedgerows) has had negative effects on their populations in agricultural landscapes [11,12]. These non-crop habitats also provide beneficial arthropods with more general benefits, in terms of shelter, nesting- and overwintering sites, that may be largely absent from modern agricultural systems [13,14,15,16].

One strategy used to ameliorate the lack of resource-rich habitat for beneficial arthropods on farmland is the establishment of ‘ecological focus areas’ (EFAs) or wildflower strips (here in ‘flower strips’) in field margins or unproductive areas nearby adjacent crops [14,17]. These habitats are often implemented as part of agri-environment schemes (AES), which offer farmers a financial incentive to adopt ‘environmentally-friendly’ management practices [18]. A large body of evidence now exists demonstrating the value of flower strips for beneficial arthropod populations and the provision of ecosystem services in adjacent farmland [19,20,21,22]. However, despite apparent synergies in the habitat requirements of pollinators and natural enemies, few studies have investigated effects of flower strips on both groups concurrently [23,24,25]. Optimising flower strips to support multiple beneficial arthropods is expected to increase their attraction for both policy-makers and farmers [26,27,28].

Where the visitation preferences of pollinators and natural enemies have been compared, there is compelling evidence of a dichotomy in the suitability of flowering plants for these groups, based on morphological incompatibilities between floral structures related to nectar accessibility (e.g., corolla depth, width) and arthropod feeding structures (e.g., tongue length) [25,29]. For example, legume-rich mixtures that are typically dominated by species whose nectar is concealed in deep corollas (e.g., *Trifolium* species), are highly attractive to eusocial bee taxa (e.g., honeybees, bumblebees), but morphologically exclude arthropods with unspecialised mouthparts. These include many important natural enemy groups (e.g., aphidophagous hoverflies, ladybird beetles), but also short-tongued bees [30]. Instead, the latter group feed on plant species that present nectar in shallow or ‘open’ structures (e.g., umbels, extra-floral nectaries) [29,31]. Therefore, inclusion of flowering plant species with opposing floral morphologies (i.e., ‘concealed’ or ‘open’ species) in seed mixtures could be a simple means of providing floral resources for multiple beneficial arthropod groups. However, few studies have considered the response of both pollinators and natural enemies to different flower mixtures [25,32], and fewer still have investigated effects on ecosystem services [33]. Thus, there is pressing need to examine the efficacy of flower strips to enhance multiple ecosystem services on farmland.

Aphids (Hemiptera: Aphididae) are major pests of many crop species, including perennial crops such as apple (*Malus domestica* Borkh.). They cause damage due to both direct effects of aphid feeding (e.g., reduced yield and tree vigour), but also through indirect effects of virus transmission by sap-sucking aphids [34]. Under favourable conditions, aphids in apple orchards are effectively controlled by a diverse range of arthropod natural enemies, many of which depend on floral resources at some point during their life cycles [35,36,37,38]. Apple is also extremely dependent on pollinators for high fruit set, particularly wild bees, that benefit from the presence of alternative floral resources in orchards [39,40,41,42]. Thus, perennial crops such as apple are well suited to the introduction of flower-rich habitats as the benefits (i.e., increased beneficial arthropod populations) could accrue and transfer across seasons, rather than just be temporarily enhanced within a single growing season [21]. However, in conventional orchards, areas between trees (alleyways) are maintained as grass-dominated swards that are frequently mown and offer little in terms of alternative floral resources for beneficial arthropods following apple blossom in spring [15].

Here, we used commercial cider apple orchards to examine the effects of perennial flower strips on the abundance and diversity of pollinators (i.e., bees) and natural enemies (i.e., aphidophagous and generalist taxa) in orchards following apple bloom, pest control services (i.e., aphid colony densities, predator-prey ratios, sentinel prey removal), and fruit production in adjacent apple trees. Specifically, we addressed the following questions: (1) Does nectar accessibility determine the diversity and abundance of pollinators and natural enemies visiting flower strips in cider apple orchards? (2) Do patterns in natural enemy abundance and diversity in flower strips correlate with the delivery of pest control services and yield in adjacent apple trees? We discuss our findings in relation to the design of flower-rich AES to boost delivery of ecosystem services in apple orchards.

## 2. Materials and Methods

### 2.1. Study Design

Field experiments were conducted in four cider apple orchards (HP Bulmer Ltd., Herefordshire, UK) and located within the same 15 × 15 km square in Herefordshire, South-West England (SO 371 434). Orchards were planted with one of three cider apple varieties (‘Gilly’, ‘Hastings’ and ‘Amanda’), and were all within the range of 4.33–16.9 ha (mean = 10.45 ha ± 2.39 SEM), of similar age (planted between 2007 and 2009), management (i.e., conventional), and spatial layout (inter-row spacing = 5.5 m; inter-tree = 2.75 m). Alleyways were maintained as a dense sward of fine-leaved grasses and herbs and were mown every other week from May until September each year.

### 2.2. Flower Strips

Based on experience from previous studies and the scientific literature, flowering plant species were selected based on nectar availability as ‘concealed’ nectar plants (14 species) or ‘open’ nectar plants (11 species) (see Table S1, Supplementary Materials). The *concealed nectar* group included those species that hold nectar in deep corollas or spurs (e.g., *Trifolium* species), which require specialised feeding structures (e.g., long proboscis) to access floral resources. The *open nectar* group included plant species that provide food rewards in flowers with short corollas (e.g., Apiaceae), or in extra-floral nectaries. Species selection was biased towards species included in existing AES [20,25,30], or used previously in experimental flower mixtures (Table S1, Appendix A1, Supplementary Materials). From these two functional groups, we devised three flower treatments including: concealed-nectar species mix, open-nectar species mix, and a ‘multi-functional’ or ‘mixed’ treatment that contained all species, but with half the amount of seed per species by weight.

Replicate plots of each flowering treatment and a grass strip control (i.e., alleyways under normal management) were marked out in orchards in April 2011. A single plot encompassed a continuous 40 m length of trees in the same row (=15 trees), and the pair of alleyways running parallel to the trees. Plots were always located at least 100 m from another, and 50 m from the orchard edge to minimise interactions between treatments and edge effects (Figure S1, Appendix A1, Supplementary Materials). In experimental plots, a pair 40 × 1 m flower strips were established down the centre of alleyways (for details on sowing protocol see Appendix A1, Supplementary Materials). Flower strips bloomed sporadically in 2011 but bloomed continuously in the following two years from late May onwards before being cut in September each year using a tractor-mounted mower to prepare alleyways for mechanical harvest. Control plots were mown on a two-weekly rotation from May to September each year.

### 2.3. Flower-Visitor Surveys

To assess response of pollinators and natural enemies in orchards to different flower mixtures, flower-visitor surveys were carried out in all plots between June and August in both years (2012 and 2013). We focused our observations on this period as floral resources for beneficial arthropods are more limited during the growing season (June until October) than prior to or during apple blossom [43]. Plots were observed 1–3 times per month by an experienced recorder, during which all flower-visiting insects were recorded by walking the full length of both alleyways at an even pace, noting the identity of insect taxa and the plant species being visited. Only taxa that could not be identified on the wing were collected and used to form a reference collection. For control plots, we focused on the central 1 m band of each alleyway to standardise the area considered in all treatments. Observations took place between 10:00 and 17:00 on calm, dry days with minimum temperatures of 13 °C if sunny, or 17 °C if overcast (in accordance with the UK Butterfly Monitoring Scheme (UKBMS website)). We considered all visiting bees (Hymenoptera: Apoidea; eusocial taxa—*Bombus* spp. and *Apis mellifera* L.; solitary taxa—Andrenidae, Melittidae, Megachilidae, Halictidae) as pollinators. Other insects contribute little to apple pollination in our study region [40,42]. As focal pests were aphids, natural enemies were separated into aphidophagous taxa: hoverflies (Diptera: Syrphidae: Syrphinae), ladybird beetles (Coleoptera: Coccinellidae), earwigs (Dermaptera: Forficulidae), and lacewings (Neuroptera: Chrysopidae); and other more generalist or unspecialised taxa, including: non-syrphid flies (Empididae, Scatophagidae, Asilidae and Tachinidae), beetles (Coleoptera: Cantharidae, Staphylinidae), bugs (Hemiptera: Anthocoridae, Miridae), and parasitic wasps (Hymenoptera: Parasitica). Bees were identified to species or aggregate groupings (e.g., *Bombus terrestris* agg.) and natural enemies to at least family level, except parasitoid wasps (super-family).

### 2.4. Apple Tree Surveys

To assess effects of flower strips on natural enemies and pest control services in adjacent apple trees, five branches (1 m in length and 1–2 m above ground) on ten trees in each plot were intensively sampled for natural enemy taxa (separated into aphidophagous and generalist species) and pest aphid colonies 1–2 times per month (June–August) in 2012 and 2013 (five surveys per year). Surveys were performed in warm, sunny conditions and the order in which plots and orchards visited was randomised. We considered an aphid colony to be any aggregation of aphids numbering more than five individuals. We focused on aphids as target pests as they were the only pest group present in all four study orchards. Aphidophagous taxa included hoverflies (egg clutches and larvae), coccinellid beetles (all life stages), earwigs (adults), and lacewings (eggs and larvae). Other natural enemy taxa included non-syrphid flies (adults), cantharid beetles (adults), bugs (nymphs and adults), and parasitoid wasps (adults). Natural enemies and aphid pests were collected using entomological net and aspirator and subsequently stored in 70% ethanol for later identification under a stereomicroscope.

### 2.5. Sentinel Egg Cards

In addition to tree surveys, batches of sterilised moth eggs (*Ephestia kuehniella*) were used as sentinel cards to measure predator activity in apple trees adjacent to experimental plots. Eggs were mounted onto special monitoring cards from Biobest (Biobest N.V., Ilse Velden 18-2260, Westerlo, Belgium), with each card holding a standardised number of eggs (238 ± 7 (SE) eggs, *n* = 20). Egg cards were put out in plots on five occasions in 2013 only between the 18 June and 9 September (1–2 times per month). For each sampling event, four cards were attached to branches on separate trees in plots at a height of 1.5 m and left for 48 h. One card per plot on each sampling date was covered in a fine nylon mesh to exclude arthropods and act as a control (*n* = 72). Cards were recaptured and then scored on a scale from 0 to 1 based on egg loss (0 = no eggs removed; 0.25 = 1–25%; 0.50 = 26–50%; 0.75 = 51–75%; 1.00 = 76–100 % removed).

### 2.6. Fruit Yield

To assess the effects of flower strips on fruit production, the number of apples was counted in September (one month prior to harvest) each year on three randomly selected branches in plots (all branches approximately 1 m in length and on separate trees). Unlike in dessert apple orchards, fruit thinning is rarely practiced in cider orchards. In 2013, up to twenty mature fruits per branch were also weighed using a digital weighing scale and measured at their widest point along their horizontal axis using callipers to assess fruit size.

### 2.7. Statistical Analyses

Data from different years were analysed separately to account for variation in flowering plant communities and environmental conditions between years. To test effects of flower strips on abundance and richness of flower-visiting pollinators (response variables: eusocial bee abundance, solitary bee abundance; richness) and natural enemies (response variables: aphidophagous taxa abundance; other taxa abundance; overall richness) in orchards, generalised linear mixed effects models (GLMMs) were fitted with negative binomial distributions (log-link function) using the R package ‘glmmADMB’ [44]. Fixed effects included treatment (factor with four levels: grass strip control, concealed nectar mix, open nectar mix, and mixed plots), sampling month (factor with three levels: June, July and August), and the interaction between treatment and sampling month. This allowed testing of continuity of treatment effects over the growing season. Random effects included plots within orchards to account for repeated measures and hierarchical experimental design.

To analyse effects of flower strips on aphid densities (response variable: number of colonies per plot—i.e., five branches on 10 trees) and natural enemies (response variables: richness per plot, abundance of aphidophagous taxa and other natural enemy taxa per plot) in adjacent apple trees, we fitted Negative binomial (NB) GLMMs. Fixed effects included treatment, sampling month and the interaction between factors, and plots nested within orchards as random effects to account for hierarchical experimental design and repeated measures. To test effects of flower strips on predation rate of exposed egg cards (excluding negative controls), we fitted a GLMM with binomial errors. Fixed effects included treatment, sampling month (June, July, August and September), and the interaction between predictor variables. Plots were nested within orchard as random effects. Effect of treatment on fruit number in plots was analysed by fitting a NB GLMM with orchard included as a random effect. Effect of treatment on size and weight of harvested apples was analysed using linear mixed effect models (LMM) in the R package ‘nlme’ [45] with individual apples nested within trees, trees nested within plots, and plots within orchards included as random effects. Fruit number per branch was included as an additional covariate to control for effects of resource allocation within trees.

Minimum adequate models were selected using a backwards stepwise procedure from the full model and likelihood ratio tests (LRT, fixed effects retained in model when *p* < 0.05). Model assumptions were checked by visually assessing residual plots as recommended in Zuur et al. [46]. All statistical analyses were performed in R ver. 3.1.3 [47].

## 3. Results

### 3.1. Flower Strips

Flower abundance was assessed each month in all plots (see Appendix A2, Supplementary materials for details). A total of 16 sown species were recorded in flower in 2012 and 2013, but only eight species flowered consistently (*T. hybridum*, *T. pratense*, *T. repens*, *L. corniculatus*, *C. montana*, *V. cracca*, *V. sativa* and *D. carota*) (Table S2, Appendix A2, Supplementary Materials). Flower spikes of white clover (*T. repens*) were common in control plots, but never in equivalent abundance to flower strips, and other unsown species were rare (<1% of total flower abundance). In either study year, mixed plots contained 60% fewer open-nectar flowers (e.g., *D. carota*) than tailored mixes (i.e., only open-nectar plants), whereas concealed-nectar species (e.g., *Trifolium* species) were found in similar abundance in both tailored and mixed plots. Flower abundance was lower in June each year compared to following months (Mean ± SE flower number per plot: 2012—June = 1250 ± 359; July = 2372 ± 528, August = 3137 ± 792; 2013—June = 570 ± 139, July = 999 ± 298, August = 823 ± 185).

### 3.2. Flower-Visitor Surveys

Over the two-year study period, 6533 flower visits by 30 distinct beneficial arthropod taxa were recorded in the study plots (for species details, see Table S3, Appendix A3, Supplementary Materials). Pollinators (bees) and natural enemies, represented 28.3 and 71.7% of visits, respectively. Pollinators (13 taxa) included eusocial bees (bumblebees—53.6% of pollinator visits; and honeybees—28.9%), and solitary bees (17.5%). Of flower-visiting natural enemies (17 taxa), 6.7% of visits were by aphidophagous taxa, including adult hoverflies, ladybird beetles and lacewings, and 93.3% by other natural enemy taxa, including hymenopteran parasitoids, non-syrphid flies, non-coccinellid beetles, and predatory bugs.

**Figure 1:**
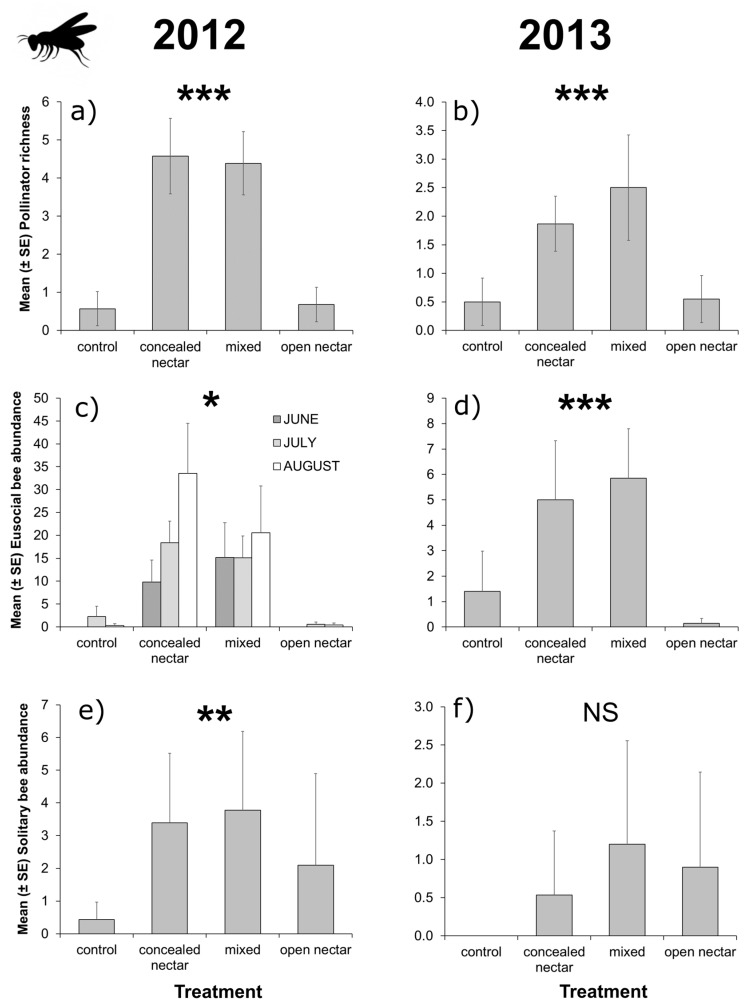
Effects of flower strips on flower-visiting pollinators in orchard alleyways. We detected significant effects of treatment (control, concealed-nectar, mixed, and open-nectar) on pollinator richness in either study year (**a**,**b**); an effect of the interaction between treatment and sampling month (June, July, and August) on eusocial bee abundance in 2012 (**c**); an effect of treatment on eusocial bee abundance in 2013 (**d**); and an effect of treatment on solitary bees in 2012 (**e**); but not 2013 (**f**). Error bars show standard errors and asterisks show level of significance (NS = *p* > 0.05, * = *p* < 0.05, ** = *p* < 0.01, *** = *p* < 0.001) reported in LRTs (see Table 1 and main text for details).

**Figure 2:**
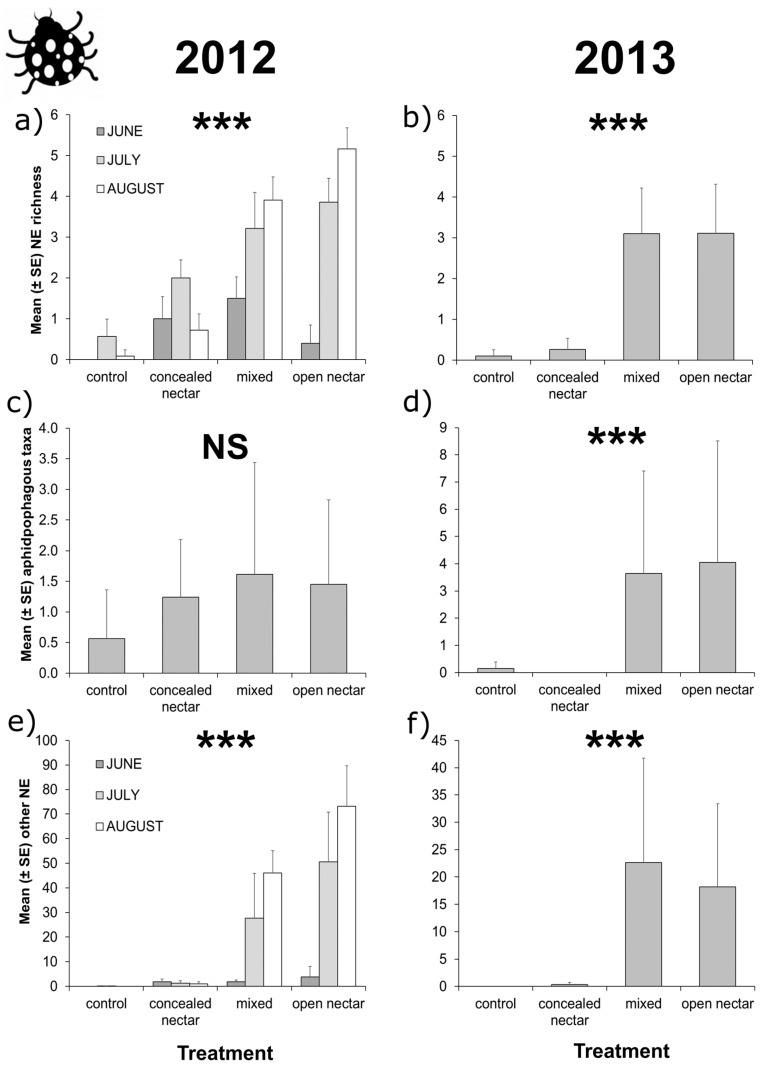
Effects of flower strips on flower-visiting natural enemies in orchard alleyways. We detected a significant effect of treatment on aphidophagous taxa in 2013 but not in 2012 (**c**,**d**); Other natural enemies and overall natural enemy richness were affected by the interaction between treatment (control, concealed-nectar, mixed, and open-nectar) and sampling month (June, July, and August) in 2012 (**a**,**e**); but by treatment only in 2013 (**b**,**f**). Error bars show standard errors and asterisks show level of significance (NS = *p* > 0.05, * = *p* < 0.05, ** = *p* < 0.01, *** = *p* < 0.001) reported in LRTs (see Table 1 and main text for details).

Overall, pollinators and natural enemies showed striking differences in flowering plant visitation patterns, as 92.6% of pollinator visits were to species included in the concealed-nectar functional group, whereas 97.2% of natural enemy visits were to flowering plants included in the open-nectar group (including visits to extra-floral nectaries of *V. sativa*). Although sub-division of pollinators into eusocial and solitary bee taxa revealed solitary bees had low preference for either functional group, with 61.7% of visits to concealed-nectar plants. In both years, pollinator richness was 70% higher in flower strips sown with concealed-nectar plants compared to control or open-nectar plots (Table 1 and Figure 1a,b). Eusocial bee abundance followed similar patterns, however, in 2012 we detected a significant interaction effect between treatment and sampling period (Table 1), as eusocial bee visitation to concealed-nectar plots peaked in August that year following intense bloom of *T. pratense* (Figure 1c). Solitary bees were more abundant in flower strips than controls but the effect of treatment was only significant in 2012 (Table 1 and Figure 1c,d). Total natural enemy richness and abundance of non-aphidophagous taxa were 90% higher in flower strips including open-nectar plants compared to other treatments (Table 1 and Figure 2a,b,e,f); although, in 2012 the interaction between treatment and sampling month had a significant effect on natural enemy flower visitation, due to low availability of open nectar plants in June that year (Figure 2a,c). Aphidophagous taxa were generally more abundant in treatments containing open-nectar plants, but the effect of treatment was only significant in 2013 (Table 1 and Figure 2c,d).

### 3.3. Apple Tree Surveys

A total of 861 aphid colonies, belonging to three species (*Aphis pomi* de Geer, *Dysaphis plantaginea* Passerini, and *Eriosoma lanigerum* Hausmann), and 1461 natural enemies (all life stages) were recorded on apple trees. Of those arthropods classed as natural enemies, 19% were aphidophagous taxa, including lacewings (eggs and larvae), ladybirds (all life stages), hoverflies (eggs and larvae), and earwigs (adults); and 81% were generalist or unspecialised (other) natural enemies, including hemipteran bugs (nymphs and adults), hymenopteran parasitoids, and non-coccinellid beetles (for species details, see Table S4, Appendix A3, Supplementary Materials).

**Figure 3:**
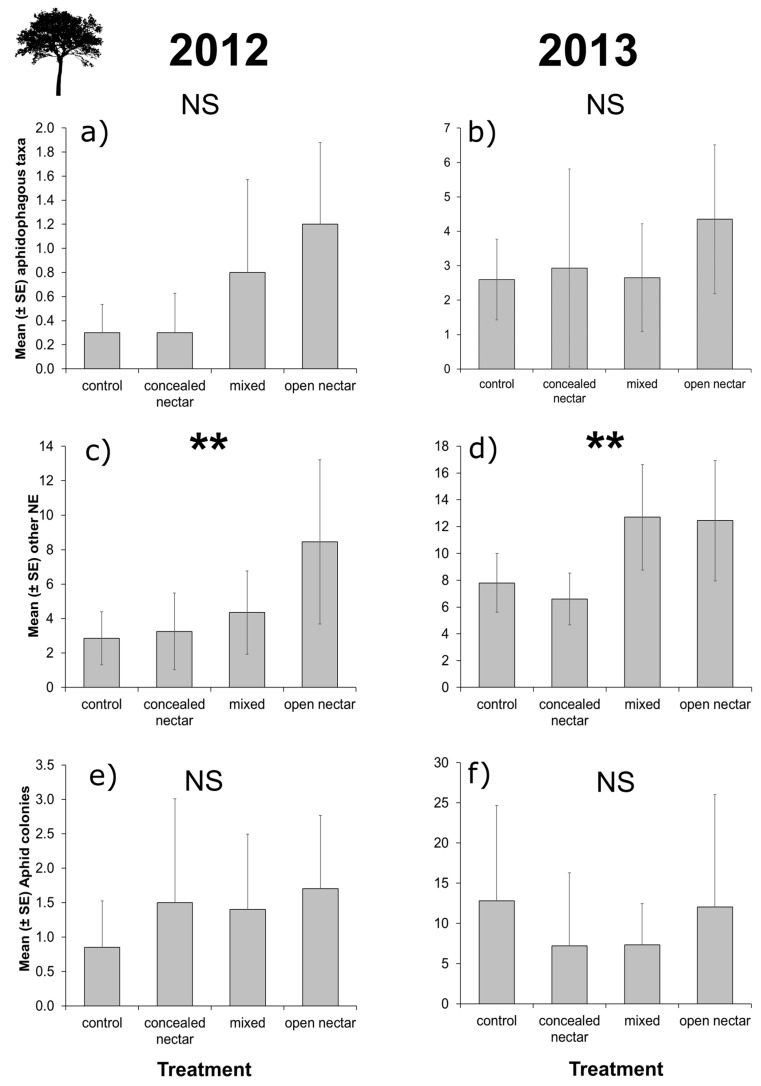
Effects of flower strips on natural enemies and aphid pests in apple trees (number of individuals/colonies per fifty branches). We detected no effect of treatment (control, concealed-nectar, mixed, and open-nectar) on aphidophagous natural enemies (hoverflies, lacewings, earwigs and ladybirds; (**a**,**b**)) or pest aphid densities in either year (**e**,**f**); but significant effects of treatment on other natural enemy abundance (non-syrphid flies, parasitoid wasps, bugs and non-coccinellid beetles; (**c**,**d**)). Error bars show standard errors and asterisks show level of significance (NS = *p* > 0.05, * = *p* < 0.05, ** = *p* < 0.01, *** = *p* < 0.001) reported in LRTs (see Table 2 and main text for details).

In both years, aphidophagous and non-aphidophagous natural enemy taxa on apple trees in plots (per fifty branches) showed clear trends for higher abundance in trees adjacent to flower strips sown with open-nectar plants (Figure 3a–d). However, the effect of treatment was only significant (*p* = 0.05) for non-aphidophagous taxa (Table 2), and aphid colony densities per plot were unaffected by flower treatment in both years (Table 2 and Figure 3e,f). Aphid predator-prey ratios (using mean values) were elevated in apple trees nearby flowering plots containing open-nectar plants compared to other treatments in 2012 (aphidophagous natural enemies per aphid colony: control = 0.35, concealed-nectar = 0.20, mixed = 0.57, open-nectar = 0.71), but were similar in all treatments in 2013 (control = 0.20, concealed-nectar = 0.41, mixed = 0.36, open-nectar = 0.36).

### 3.4. Sentinel Egg Cards

**Figure 4:**
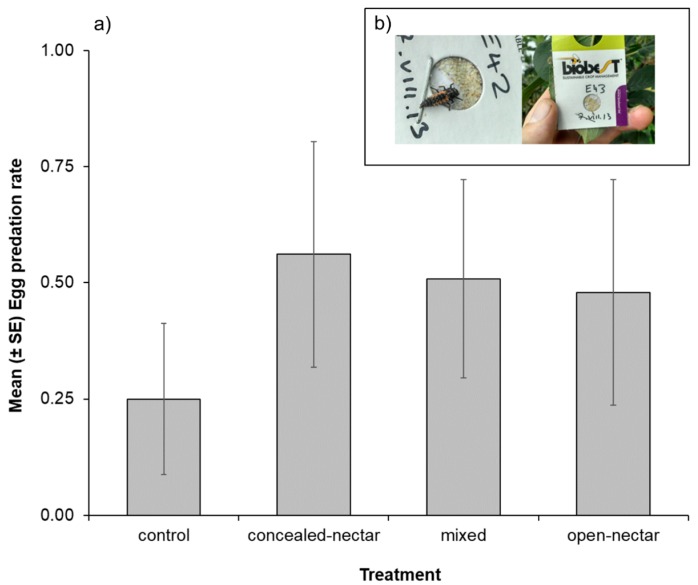
Mean (±SE) predation rate (0 = no predation and 1 = complete removal) of sentinel moth eggs in apple trees adjacent to different flower mixtures and control plots in 2013. (**a**). Inset photographs (**b**) show coccinellid larva feeding on eggs and example of card in apple foliage.

Several natural enemy taxa were observed attacking exposed egg cards, including predatory hemipterans, coccinellid adults and larvae, and neuropteran larvae (Figure 4, inset). Comparison of data from negative controls (natural enemies excluded) confirmed egg losses were due to arthropod predator activity (Mean ± SE egg losses: negative controls = 0.03 ± 0.01, *n* = 75; exposed cards = 0.44 ± 0.03, *n* = 225). Egg predation was enhanced in all plots with sown flower strips compared control plots (Table 2 and Figure 4), with predation rates increasing by up to 55% in flowering plots.

### 3.5. Fruit Yield

**Figure 5:**
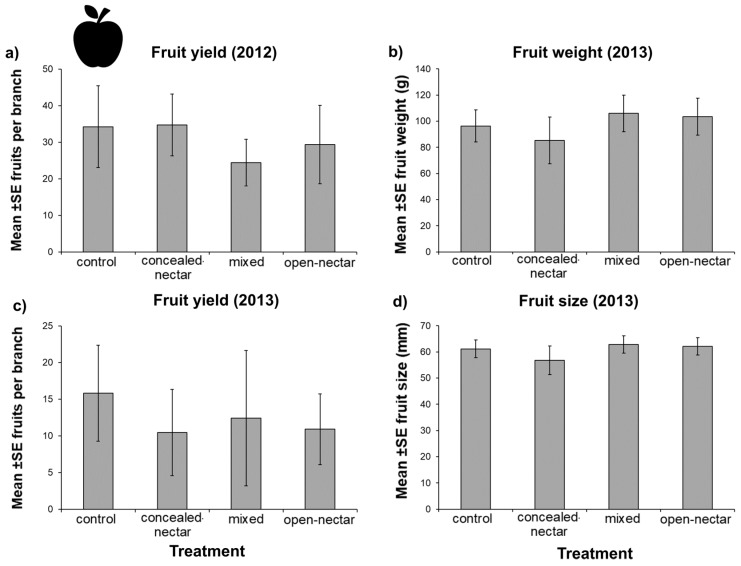
Effects of flower strips on fruit yield and fruit quality (size and weight) in orchards. We detected no effect of treatment (control, concealed-nectar, mixed, and open-nectar) on fruit yield in either study year (**a**,**c**); or on fruit size (**b**) and weight (**d**) in 2013. Error bars show standard errors.

Fruit number per branch varied between years, with counts in 2013 around 50% reduced relative to 2012. Fruit number was not significantly affected by treatment in either year (Table 3), although, in 2012 the effect of treatment was marginally significant on fruit number (Table 3), being lowest in mixed plots (Figure 5). We detected no effect of treatment on either size or weight of harvested fruit in 2013 (Table 3 and Figure 5).

## 4. Discussion

Flower-rich agri-environment schemes (AES) aim to mitigate biodiversity losses and improve multiple ecosystem functions on farmland. However, while there already exists a large body of work demonstrating their value for single ecosystem functions [19,20,21], evidence on their capacity to support multiple ecosystem services (e.g., pollination and pest control) in crops remains limited. Here, we demonstrate that careful selection of plant species based on floral structures that determine nectar accessibility and insect flower visitation patterns can be used to design flower strips that attract both pollinators and natural enemies in apple orchards, and enhance natural enemy activity in adjacent apple trees. However, we found no evidence that enhanced natural enemy communities improved control of aphid pests or fruit yield in studied orchards. We discuss the implications of our findings for the design of AES in perennial orchards crops.

**Question** **1.** Do floral traits determine the diversity and abundance of pollinators and natural enemies in cider apple orchards?

Positive relationships between plant and insect diversity are common in flower-visitor communities [2]. It is now clear that underlying these trends are changes in the diversity of morphological or physiological characteristics of flowering plant species (i.e., functional traits) that act as signals or barriers for feeding by different animal species (e.g., flower colour, shape, volatile profile, resource quantity/quality, bloom period), rather than changes in species diversity per se [48]. Thus, plant species that share floral traits are expected to attract similar subsets of flower visitors, and can be considered as a single functional group [25]. In an applied context, this ‘trait-matching’ approach can be used to design ‘tailored’ flower strips that target different subsets of beneficial arthropods (e.g., pollinators and natural enemies) and promote the delivery of ecosystem services in adjacent crop plants.

Here, in concordance with expectations, we found bees predominantly visited the flowers of plant species included in the concealed-nectar functional group, i.e., species that store nectar in long corollae or spurs, whereas natural enemies mainly visited plants included in the open-nectar group, i.e., species that present nectar in shallow or open structures. This reflects the fact that many natural enemies have unspecialised (i.e., short) mouthparts that restrict feeding on concealed-nectar plants, the preferred food plants of many bee pollinators [25,29,31]. Thus, when plant functional groups were presented singularly, flower strips were visited either by pollinators or natural enemies, but when mixes were combined (mixed or ‘multi-functional’ treatment), they attracted both groups concurrently, and in most cases in similar abundance to preferred targeted mixes. Importantly, these patterns remained consistent over the two-year study period, even though a high rate of turnover in flowering plant species was observed between years, reaffirming the value of a functional trait-based approach to plant species selection in flower strips.

In many countries, AES options available to farmers to boost beneficial arthropods comprise of simple mixes of ‘four or five nectar-rich plants’ from the Fabaceae (e.g., England HF4 pollen and nectar mix) [30], analogous to the ‘concealed-nectar’ treatment. Our data suggest that such mixtures provide little in terms of floral resources for pest natural enemies [30], and inclusion of open-nectar plants in mixes offers a simple means to provide floral resources for both pollinators and natural enemies. Although, with the exception of *Trifolium* species specialists (e.g., *Melitta leporina* Panzer), and bivoltine taxa that preferentially visited late season open-nectar plants (e.g., *Andrena minutula* Kirby), solitary bees were infrequent visitors to flower strips compared to eusocial bees (honeybees and bumblebees). This also held for important apple pollinators, such as *Osmia bicornis* L. and large-bodied *Andrena* species, that have short flight periods (March–July) [43,49]. Thus, the selected floral prescriptions, while being highly attractive to eusocial bees, may be of limited value for key apple pollinator taxa, because of temporal incompatibilities between flight periods and peak bloom of included plant species. This may explain why these mixtures failed to enhance pollination services in studied orchards when compared to orchards without flower strips [40].

**Question** **2.** Does natural enemy visitation to flower strips correlate with the delivery of pest control services and yield in adjacent apple trees?

Flower strips will provide clearest benefit to pest control services if they have positive impacts on the fitness of functionally-important natural enemies, ideally without supporting damaging pest species [29,50]. Therefore, plants selected to support pest control should not only attract flower-feeding natural enemies, but also provide measurable fitness benefits in terms of improved longevity and/or fecundity that leads to increases in their population size and function (i.e., predation) in adjacent crops. Some aphid pests in apple orchards can benefit from flowering vegetation if it includes secondary host plant species (e.g., *D. plantaginea* on *Plantago lanceolata* L.), but this species was not included in seed mixtures.

Although we did not directly measure impacts on natural enemy fitness, we detected clear trends for higher densities of natural enemies in apple trees near those flower strips that contained open-nectar species. This indicates that the inclusion of plants with shallow or open nectaries not only attracted or retained natural enemies, but likely also provided fitness benefits compared to plots without open-nectar plants. Furthermore, reduced natural enemy densities in mixed plots relative to the open-nectar plots suggested that fitness benefits were directly related to densities of accessible flowering plant species, not overall flower abundance in plots [51]. Therefore, increasing the diversity of flower structures in flowering strips may involve trade-offs between ecosystem services, because of the non-overlapping plant-feeding preferences of natural enemies and pollinators [52]. We did not detect similar effects on pollinator visitation as concealed-nectar plants were found in equivalent abundance in different treatments, despite 50% reduced seed in mixed plots.

In contrast to natural enemy densities, predation of sentinel prey was elevated in all flowering plots relative to controls. It is possible that ‘non-target’ mixes also provided benefits to natural enemies, such as alternate prey or shelter for generalist predators (e.g., anthocorid bugs, earwigs) that have lower dependence on floral resources [37,38,53], but that may have been under sampled during flower-visitor and apple tree surveys (e.g., small body size or nocturnal activity period). Furthermore, predation on sentinel egg cards remained high up to three weeks after the flowering strips had been mown, which suggested a more permanent, population-level increase in natural enemy densities in alleyways with flower strips, rather than a transient displacement or aggregation of individuals in trees during flowering periods [54].

Despite positive effects on natural enemy densities and sentinel prey removal, we found no clear evidence that flower strips affected aphid colony densities, fruit number, or quality of harvested fruit (weight and size) in plots. The absence of a yield effect in studies of flower strips in orchards is not uncommon, as Simon et al. (2010) found in a review of 30 studies that just under half showed either no effect, or even negative effects on fruit yields [55].

Possible explanations for discrepancies between the responses of natural enemies, pests, and yield to flower strips are numerous. The most obvious explanation for the absence of effect on aphid pest control was that aphidophagous taxa (e.g., lacewings, coccinellids, hoverflies and earwigs) responded weakly to flower strips compared to other natural enemy taxa. This is probably due to lower dependence of some aphidophagous taxa (e.g., coccinellid beetles, earwigs) on flowering plants compared to other natural enemy taxa [15,56]. Although results from sentinel prey assays suggested that natural enemy activity was enhanced in all flowering plots irrespective of plant species composition. However, caution is required as sentinel prey removal rates may not necessarily reflect pest control services if species attacking eggs differ from those attacking pest species. Alternatively, natural enemy increases may have come too late to alter pest-yield dynamics in orchards, as many aphid pests attack apple trees from late spring onwards [57]. Thus, one solution could be to increase the number of early-flowering plants in seed mixtures, particularly species that provide nectar and pollen for hoverflies, as they are considered to be highly effective early season predators of aphids in apple orchards [36]. Nevertheless, high predator densities at the end of the season can reduce the following year’s aphid pest burden through predation of dormant life history stages (e.g., egg masses) [58], but such effect may only be revealed over longer time periods than considered in the present study [59]. Yet, trees are also responding to attacks by non-aphid pests (e.g., apple sawfly *Hoplocampa testudinea* Klug, apple blossom weevil *Anthonomus pomorum* L., codling moth *Cydia pomonella* L.), many of which may be poorly controlled by natural enemies, as well as changes in nutrient/water availability, pollination services and climate. Therefore, positive effects of an enhanced natural enemy community may be blurred by other factors that limit yield in apple orchards [60,61,62].

Yet, perhaps the most important factor in explaining the lack yield effect was the use of pesticides on studied farms. All farms were sprayed prior to and immediately after blossom to control damaging pest species that are not readily controlled by natural enemies (e.g., *A. pomorum* and *H. testudinea*) [15]. Thus, while pesticide applications probably ensured that pest densities (including aphids) were kept below economic thresholds, they more than likely decimated emerging natural enemies and limited the transfer of benefits from flower strips between growing seasons. Future studies should look at impacts of flower strips across a gradient of agrochemical usage to assess whether natural enemies can replace or improve on ecosystem functions currently provided by agrochemical inputs in orchards under conventional management [8].

## 5. Conclusions

In summary, we show that with careful selection of plant species, flowering strips can provide floral resources for both pollinators and natural enemies in orchards, and enhance predator activity in adjacent apple trees. However, further work is required to optimise the design and management of flowering strips to include a greater number of early-flowering plant species for both efficient apple pollinator taxa (e.g., spring-flying solitary bees) and functionally-important natural enemies of aphids in orchards, and across a gradient of pesticide use to fully examine their potential to replace ecosystem functions presently provided by agrochemical inputs in conventionally-managed orchards. Only through such means can we truly enable an ‘ecological intensification’ of orchard farming practices, that benefits both biodiversity and fruit production in orchards.

## Figures and Tables

**Table 1:** Summary of minimum adequate models selected by inference on likelihood ratio tests. Effects of treatment (factor with four levels: control, concealed-nectar mix, mixed plots, and open-nectar mix), sampling month (factor with three levels: June, July and August), and the interaction between treatment (T) and month (M) on pollinator richness, pollinator abundance (eusocial bees and solitary bees; flower strips only), aphidophagous natural enemy abundance, other natural enemy abundance and natural enemy richness in orchard alleyways. Degrees of freedom (d.f.), test statistics (LRT) and *p*-values from likelihood ratio tests are shown. *p*-values of fixed effects included in final models are presented in bold (*p* < 0.05).

		2012			2013		
		Within Flower Strip			Within Flower Strip		
Response	Predictor	d.f.	LRT	*p*	d.f.	LRT	*p*
**Pollinators**							
Richness	Treatment	3	34.23	**<0.001**	3	17.44	**0.001**
	Month	2	5.63	0.060	2	9.13	**0.010**
	T × M	6	9.67	0.139	6	7.72	0.259
Eusocial bees	Treatment				3	22.51	**<0.001**
	Month				2	18.37	**<0.001**
	T × M	6	15.34	**0.018**	6	6.79	0.341
Solitary bees	Treatment	3	13.96	**0.003**	3	7.02	0.071
	Month	2	8.98	**0.011**	2	10.79	**0.005**
	T × M	6	8.49	0.204	6	4.85	0.564
**Natural enemies**							
Richness	Treatment				3	35.22	**<0.001**
	Month				2	23.14	**<0.001**
	T × M	6	14.51	**0.024**	6	3.91	0.689
Aphidophagous taxa	Treatment	3	3.27	0.352	3	18.47	**<0.001**
	Month	2	21.46	**<0.001**	2	24.82	**<0.001**
	T × M	6	2.27	0.893	6	9.53	0.146
Other taxa	Treatment				3	28.00	**<0.001**
	Month				2	8.42	**0.015**
	T × M	6	40.02	**<0.001**	6	8.74	0.189

**Table 2:** Summary of minimum adequate models selected by inference on likelihood ratio tests. Effects of treatment (factor with four levels: control, concealed-nectar mix, mixed plots, and open-nectar mix), sampling month (factor with three levels *: June, July and August), and the interaction between treatment (T) and month (M) on natural enemy richness, abundance of aphidophagous taxa, non-aphidophaous natural enemy taxa, aphid colony densities, and egg card predation within adjacent apple trees in each year. Arthropods sampled on ten trees (five branches per tree) per plot on five separate occasions each year in four orchards. Degrees of freedom (d.f.), test statistics (LRT) and *p*-values from likelihood ratio tests are shown. *p*-values of fixed effects included in final models are presented in bold (*p* < 0.05).

		2012			2013		
		Within Crop			Within Crop		
Response	Predictor	d.f.	LRT	*p*	d.f.	LRT	*p*
**Natural enemies**							
Richness	Treatment	3	2.40	0.493	3	2.65	0.449
	Month	2	34.80	**<0.001**	2	0.60	0.742
	T × M	6	2.32	0.888	6	1.09	0.982
Aphidophagous taxa	Treatment	3	6.35	0.096	3	1.93	0.587
	Month	2	18.29	**<0.001**	2	5.61	0.060
	T × M	6	6.16	0.406	6	11.25	0.081
Other taxa	Treatment	3	13.87	**0.003**	3	12.77	**0.005**
	Month	2	83.47	**<0.001**	2	3.33	0.189
	T × M	6	5.01	0.543	6	7.76	0.256
**Pest control**							
Aphid colonies	Treatment	3	1.54	0.672	3	3.88	0.275
	Month	2	18.97	**<0.001**	2	55.28	**<0.001**
	T × M	6	9.37	0.154	6	5.39	0.495
Egg cards	Treatment	-	-	-	3	9.54	**0.023**
	Month *	-	-	-	3	77.71	**<0.001**
	T × M	-	-	-	9	15.58	0.076

* Egg card data collected over four months (June–September 2013).

**Table 3:** Summary of minimum adequate models selected by inference on likelihood ratio tests. Effect of treatment (factor with four levels: control, concealed-nectar mix, mixed plots, and open-nectar mix) on fruit number per branch (2012 and 2013; 3 branches per plot, four plots per orchard, *n* = 192), and size (mm) and weight (g) of harvested fruit (2013) in orchards. Degrees of freedom (d.f.), test statistics (LRT) and *p*-values from likelihood ratio tests are shown.

	Treatment		
Response Variable	LRT	d.f.	*p*
2012			
Fruit number	7.01	3	0.071
2013			
Fruit number	4.65	3	0.200
Fruit size (mm)	5.09	3	0.165
Fruit weight (g)	5.94	3	0.114

## Supplementary Materials

The following are available online at www.mdpi.com/2075-4450/8/3/101/s1, Figure S1: Experimental plot design. Grey lines represent tree rows (fifteen trees per plot), Table S1: Plant species composition, (H) Habit (P—Perennial, A—Annual or B—Biennial), Trait group (CNP = concealed nectar, ONP = open nectar), percentage contribution (by weight) in each treatment (MF = Multi-functional) and literature source, Table S2: Floral unit classification (FH = flower head), median flower number per unit, median flower width (mm) and floral area (mm) for all flowering species, Table S3: Insect visitor taxa and number of visits each year during observations (*n* = No. of replicates per year) of experimental plots (con = control, cnp = concealed-nectar, mix = mixed treatment, onp = open-nectar). Visiting insect species are grouped into pollinators (POL), aphidophagous predators (APH) and generalist natural enemies (GEN), Table S4: Aphid colony densities and natural enemy taxa (abundance and richness) recorded in adjacent apple trees each year during tree surveys (*n* = No. of repeat surveys) of experimental plots (con = control, cnp = concealed-nectar, mix = mixed treatment, onp = open-nectar). Insect taxa are grouped into crop pests (CP), aphidophagous predators (APH) and generalist natural enemies (GEN). See main text for details on collected life history stages.
