# Supplementary material for: Getting More Power from Your Flowers: Multi-Functional Flower Strips Enhance Pollinators and Pest Control Agents in Apple Orchards
Source: Insects. 2017 Sep 20;8(3):101. doi: 10.3390/insects8030101 (PMC5620721; doi:10.3390/insects8030101)
Supplement: Supplementary file 1 [file insects-08-00101-s001.docx]

**Supplementary Materials**

*A1. Flower Mixes and Sowing Protocol*

**Table S1.** Plant species composition, (H) Habit (P—Perennial, A—Annual or B—Biennial), Trait group (CNP = concealed nectar, ONP = open nectar), percentage contribution (by weight) in each treatment (MF = Multi-functional) and literature source.

|  | **Plants Latin Name** | **Common Name** | **H** | **Trait** | **% Contribution to Mix (Seed Weight)** | | | **Cited in** |
| --- | --- | --- | --- | --- | --- | --- | --- | --- |
|  |  |  |  |  | **ONP** | **CNP** | **MF** |  |
| *Herbs* | *Centaurea montana* | Perennial cornflower | P | CNP | - | 8.2 | 4.1 | [1] |
|  | *Cichorium intybus* | Chicory | P | CNP | - | 5.5 | 2.7 | [2] |
|  | *Hypericum perforatum* | St. John's wort | P | CNP | - | 2.7 | 1.4 | [2] |
|  | *Knautia arvensis* | Field scabious | P | CNP | - | 8.2 | 4.1 | [2,3] |
|  | *Lamium purpureum* | Red deadnettle | A | CNP | - | 2.8 | 1.4 | [1] |
|  | *Lotus corniculatus* | Bird's foot trefoil | P | CNP | - | 8.2 | 4.1 | [4] |
|  | *Medicago sativa* | Alfalfa | P | CNP | - | 2.8 | 1.4 | [5] |
|  | *Phacelia tanacetifolia* | Phacelia | A | CNP | - | 5.5 | 2.7 | [6] |
|  | *Trifolium pratense* | Red clover | P | CNP | - | 2.7 | 1.4 | [4] |
|  | *Trifolium hybridum* | Alsike clover | P | CNP | - | 2.7 | 1.4 | [4] |
|  | *Primula veris* | Cowslip | P | CNP | - | 2.7 | 1.4 | [7] |
|  | *Clinopodium vulgare* | Wild basil | P | CNP | - | 2.7 | 1.4 | [8] |
|  | *Trifolium repens* | White clover | P | CNP | - | 2.7 | 1.4 | [4] |
|  | *Vicia cracca* | Tufted vetch | P | CNP | - | 8.2 | 4.1 | [9] |
|  | *Fagopyrum esculentum* | Buckwheat | A | ONP | 7.2 | - | 3.6 | [10] |
|  | *Achillea millefolium* | Yarrow | P | ONP | 3.6 | - | 1.8 | [11] |
|  | *Lobularia maritima* | Sweet alyssum | A | ONP | 1.0 | - | 0.5 | [12] |
|  | *Foeniculum vulgare* | Fennel | A | ONP | 7.2 | - | 3.6 | [11] |
|  | *Daucus carota* | Wild carrot | B | ONP | 3.6 | - | 1.8 | [13] |
|  | *Pastinaca sativa* | Wild parsnip | A | ONP | 3.6 | - | 1.8 | [2] |
|  | *Ammi majus* | Queen Anne's lace | A | ONP | 7.2 | - | 3.6 | [14] |
|  | *Coriandrum sativum* | Coriander | A | ONP | 7.2 | - | 3.6 | [15] |
|  | *Tanacetum vulgare* | Tansy | P | ONP | 3.5 | - | 1.7 | [16] |
|  | *Anethum graveolens* | Dill | A | ONP | 3.6 | - | 1.7 | [17] |
|  | *Vicia sativa* | common vetch | P | ONP | 10.8 | - | 5.4 | [18] |
|  | *Conopodium majus* | Pignut | P | ONP | 7.2 | - | 3.6 | - |
| *Grasses* | *Cynosurus cristatus* | Crested dog's tail |  |  | 8.6 | 8.6 | 8.6 |  |
|  | *Festuca rubra* | Slender red fescue |  |  | 6.8 | 6.8 | 6.8 |  |
|  | *Agrostis capillaries* | Brown bent |  |  | 10.3 | 10.3 | 10.3 |  |
|  | *Poa pratensis* | Smooth meadow grass |  |  | 8.6 | 8.6 | 8.6 |  |

In April 2011, plots selected for flowering treatments were first treated with herbicide (Round-Up 4l/ha), rotovated, and then sown with mixtures by hand down the central band (1 m) of each alleyway at a rate of 30kg/ha (for percentage contribution of individual species, see Table 1). After poor initial establishment, one orchard was resown successively in September 2011 and March 2012, and was therefore not considered during summer 2011. In mid-August each year, flowering strips were mown and the cuttings removed to prepare the alleyways for mechanical harvesting. Control plots were mown on a two-weekly rotation from May until September each year. To control the growth of grasses in sown strips, a graminicide, Fluazifop-P-butyl (Fusilade Max 125 g l^−1^ EC), was applied to all strips in March 2012 and April 2013 using a knapsack sprayer at a rate of 1 l/ha to 200 l of water, half the recommended rate in order to suppress, but not eliminate grass species [19].


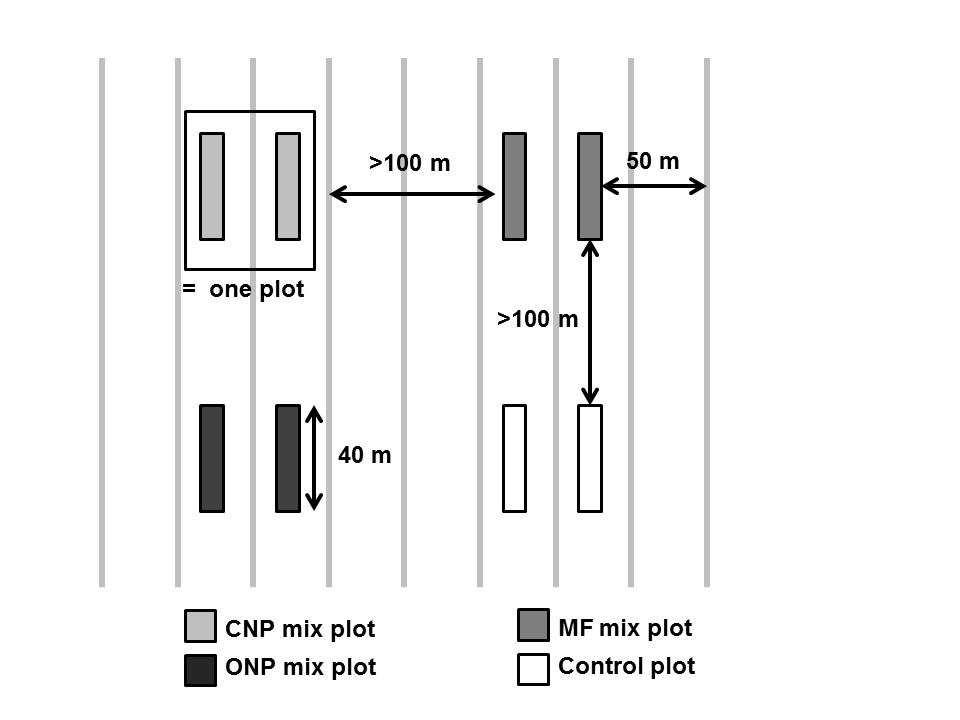


**Figure S1.** Experimental plot design.

*A2. Estimating Flower Abundance*

To characterise flowering plant communities within plots, the abundance and species richness of flowers in both alleyways was recorded several times each study year (prior to every flower-visitor survey). During flower surveys, inflorescences of different species were considered as equivalent units of each other, e.g., a flower spike of white clover (T. repens) was equal to an umbel of wild carrot (D. carota). During each sampling event, the number of floral units of each species, including unsown ‘volunteer’ species, was counted in ten randomly placed 1 × 1 m quadrats (=five per strip/alleyway). Abundance counts were then weighted by measurements of flower size (taken from Rose 2006; [20]) to account for differences in floral display size among species (e.g., a head of T. pratense = 30 mm, an umbel of D. carota = 50 mm) (see Table A2 for more details). For three of the 25 species (*Phacelia tanacetifolia*, *Ammi majus* and *Coriandrum sativum*), we used data from other similar species to calculate floral area, as these species were not included in Rose (2006). Adjusted counts were then summed across quadrats to give scaled flower abundance per plot for each sampling round. Richness in plots was recorded as the number of species recorded across all quadrats.

**Table S2.** Floral unit classification (FH = flower head), median flower number per unit, median flower width (mm) and floral area (mm) for all flowering species.

| **Plant Species** | **Floral Unit** | **Med. Fl.** | **Med. Width (mm)** | **Floral Area (mm)** | **Substitute** |
| --- | --- | --- | --- | --- | --- |
| *Lamium purpureum* | group of fl. | 6.5 | 12.5 | 81.25 |  |
| *Primula veris* | group of fl. | 20 | 9 | 180 |  |
| *Centaurea montana* | 1 FH | 1 | 70 | 70 |  |
| *Hypericum perforatum* | 1 FH | 1 | 20 | 20 |  |
| *Vicia cracca* | 1 raceme | 25 | 10 | 250 |  |
| *Trifolium pratense* | 1 FH | 1 | 30 | 30 |  |
| *Medicago sativa* | 1 raceme | 1 | 40 | 40 |  |
| *Trifolium hybridum* | 1 FH | 1 | 25 | 25 |  |
| *Trifolium repens* | 1 FH | 1 | 25 | 25 |  |
| *Phacelia tanacetifolia* | 1 raceme | 25 | 10 | 250 | * V. cracca |
| *Lotus corniculatus* | group of fl. | 5 | 15 | 75 |  |
| *Knautia arvensis* | 1 FH | 1 | 35 | 35 |  |
| *Clinopodium vulgare* | group of fl. | 6.5 | 17.5 | 113.75 |  |
| *Cichorium intybus* | 1 FH | 1 | 32.5 | 32.5 |  |
| *Anethum graveolens* | 1 umbel | 1 | 60 | 60 |  |
| *Vicia sativa* | 1 pair of fl. | 2 | 20 | 40 |  |
| *Coriandrum sativum* | 1 umbel | 1 | 50 | 50 | * A.millefolium |
| *Achillea millefolium* | 1 umbel | 1 | 50 | 50 |  |
| *Conopodium majus* | 1 umbel | 1 | 50 | 50 |  |
| *Ammi majus* | 1 umbel | 1 | 50 | 50 | * A.millefolium |
| *Lobularia maritima* | 1 raceme | 15 | 6 | 90 |  |
| *Daucus carota* | 1 umbel | 1 | 50 | 50 |  |
| *Pastinaca sativa* | 1 umbel | 1 | 75 | 75 |  |
| *Fagopyrum esculentum* | group of fl. | 1 | 75 | 75 |  |
| *Foeniculum vulgare* | 1 umbel | 1 | 60 | 60 |  |
| *Taraxacum officinale* | 1 FH | 1 | 40 | 40 |  |
| *Ranunculus repens* | 1 FH | 1 | 30 | 30 |  |
| *Prunella vulgaris* | 1 FH | 1 | 35 | 35 |  |

*A3. Insect communities in flower strips and apple trees*

**Table S3.** Insect visitor taxa and number of visits each year during observations (*n* = no. of replicates per year) of experimental plots (con = control, cnp = concealed-nectar, mix = mixed treatment, onp = open-nectar). Visiting insect species are grouped into pollinators (POL) and natural enemies (NE).

| **Taxa** | **Func.** | **2012** | | | | **2013** | | | | **Total** |
| --- | --- | --- | --- | --- | --- | --- | --- | --- | --- | --- |
|  |  | **con (*n* = 30)** | **cnp (*n* = 33)** | **mix (*n* = 31)** | **onp (*n* = 31)** | **con (*n* = 20)** | **cnp (*n* = 15)** | **mix (*n* = 20)** | **onp (*n* = 20)** |  |
| Apidae | | | | | | | | | | |
| *Apis mellifera* | POL | 29 | 267 | 200 | 3 | 8 | 11 | 15 | 0 | **533** |
| *Bombus hortorum* | POL | 0 | 78 | 41 | 0 | 0 | 0 | 1 | 0 | **120** |
| *B. hypnorum* | POL | 0 | 0 | 0 | 0 | 0 | 0 | 1 | 0 | **1** |
| *B. lapidarius* | POL | 1 | 92 | 128 | 0 | 9 | 5 | 28 | 0 | **263** |
| *B. pascuorum* | POL | 0 | 211 | 109 | 9 | 1 | 42 | 48 | 1 | **421** |
| *B. pratorum* | POL | 0 | 2 | 0 | 0 | 0 | 0 | 0 | 0 | **2** |
| *B. rupestris* | POL | 0 | 0 | 3 | 0 | 0 | 0 | 1 | 0 | **4** |
| *B. terrestris* | POL | 5 | 72 | 48 | 1 | 10 | 17 | 24 | 2 | **179** |
| Halictidae | | | | | | | | | | |
| *Lasioglossum* spp. | POL | 3 | 9 | 23 | 1 | 0 | 0 | 10 | 3 | **49** |
| *Halictus rubicundus* | POL | 0 | 10 | 3 | 1 | 0 | 2 | 7 | 0 | **23** |
| Andrenidae | POL | 3 | 12 | 32 | 47 | 0 | 0 | 2 | 15 | **111** |
| Melittidae | POL | 2 | 72 | 44 | 1 | 0 | 5 | 5 | 0 | **129** |
| Megachilidae | POL | 0 | 5 | 5 | 0 | 0 | 1 | 0 | 0 | **11** |
| Parasitica | NE | 0 | 7 | 465 | 783 | 0 | 0 | 222 | 260 | **1737** |
| Syrphidae | | | | | | | | | | |
| *Dasysyrphus* sp. | NE | 0 | 0 | 0 | 0 | 0 | 0 | 2 | 0 | **2** |
| *Episyrphus sp.* | NE | 4 | 25 | 27 | 19 | 3 | 0 | 27 | 25 | **130** |
| *Eupeodes sp.* | NE | 1 | 0 | 3 | 1 | 0 | 0 | 1 | 0 | **6** |
| *Platycheirus* sp. | NE | 7 | 7 | 6 | 1 | 0 | 0 | 5 | 6 | **32** |
| *Sphaeorphoria* sp. | NE | 0 | 0 | 2 | 2 | 0 | 0 | 0 | 8 | **12** |
| *Syrphus* sp. | NE | 5 | 8 | 11 | 5 | 0 | 0 | 31 | 29 | **89** |
| Asilidae | NE | 0 | 0 | 0 | 1 | 0 | 0 | 0 | 0 | **1** |
| Empididae | NE | 0 | 1 | 4 | 1 | 0 | 3 | 9 | 4 | **22** |
| Scatophagidae | NE | 1 | 13 | 96 | 112 | 0 | 0 | 1 | 5 | **228** |
| Tachinidae | NE | 0 | 2 | 49 | 136 | 0 | 1 | 385 | 713 | **1286** |
| Cantharidae | NE | 0 | 20 | 285 | 564 | 0 | 0 | 102 | 98 | **1069** |
| Coccinellidae | NE | 0 | 1 | 1 | 13 | 0 | 0 | 7 | 9 | **31** |
| Staphylinidae | NE | 0 | 0 | 1 | 0 | 0 | 0 | 0 | 3 | **4** |
| Chrysopidae | NE | 0 | 0 | 0 | 4 | 0 | 0 | 0 | 4 | **8** |
| Anthocoridae | NE | 0 | 0 | 4 | 3 | 0 | 1 | 8 | 4 | **20** |
| Capsidae | NE | 0 | 0 | 2 | 6 | 0 | 0 | 1 | 1 | **10** |
| **Richness** | **-** | **11** | **20** | **25** | **22** | **5** | **10** | **24** | **18** | **30** |
| **TOTAL** | **-** | **61** | **914** | **1592** | **1714** | **31** | **88** | **943** | **1190** | **6533** |

**Table S4.** Aphid colony densities and natural enemy taxa (abundance and richness) recorded in adjacent apple trees each year during tree surveys (*n* = no. of repeat surveys) of experimental plots (con = control, cnp = concealed-nectar, mix = mixed treatment, onp = open-nectar). Insect taxa are grouped into crop pests (CP) and natural enemies (NE).

|  | | **2012** | | | | **2013** | |  | | | |
| --- | --- | --- | --- | --- | --- | --- | --- | --- | --- | --- | --- |
| **Taxa** | **Function** | **con (n = 20)** | **cnp (n = 20)** | **mix (n = 20)** | **onp (n = 20)** | **con (n = 20)** | **cnp (n = 15)** | **mix (n = 20)** | **onp (n = 20)** | | **Total** |
| Hemiptera | | | | | | | | | | | |
| Aphididae (col.) | CP | 17 | 30 | 28 | 34 | 256 | 108 | 147 | 241 | | 861 |
| Hymenoptera |  |  |  |  |  |  |  |  |  | |  |
| Parasitica | NE | 41 | 37 | 40 | 21 | 25 | 18 | 42 | 43 | | 267 |
| Diptera |  |  |  |  |  |  |  |  |  | |  |
| Asilidae | NE | 1 | 2 | 2 | 0 | 1 | 0 | 1 | 0 | | 7 |
| Dolichopodidae | NE | 0 | 0 | 1 | 0 | 21 | 13 | 34 | 76 | | 145 |
| Phoridae | NE | 2 | 0 | 3 | 0 | 0 | 0 | 0 | 0 | | 5 |
| Rhagionidae | NE | 5 | 0 | 0 | 1 | 1 | 0 | 1 | 3 | | 11 |
| Scathophagidae | NE | 12 | 20 | 42 | 120 | 8 | 6 | 9 | 29 | | 246 |
| Syrphidae | NE | 0 | 4 | 5 | 10 | 10 | 15 | 6 | 4 | | 54 |
| Tachinidae | NE | 1 | 1 | 1 | 0 | 1 | 1 | 2 | 3 | | 10 |
| Coleoptera | | | | | | | | | | | |
| Cantharidae | NE | 8 | 4 | 3 | 8 | 20 | 14 | 58 | 19 | | 134 |
| Coccinellidae | NE | 5 | 0 | 2 | 4 | 13 | 18 | 24 | 31 | | 97 |
| Hemiptera | | | | | | | | | | | |
| Anthocoridae | NE | 0 | 11 | 3 | 4 | 49 | 25 | 75 | 61 | | 228 |
| Miridae | NE | 9 | 9 | 10 | 7 | 28 | 19 | 22 | 24 | | 128 |
| Reduviidae | NE | 1 | 0 | 0 | 2 | 0 | 0 | 0 | 0 | | 3 |
| Neuroptera | | | | | | | | | | | |
| Chrysopidae | NE | 1 | 2 | 5 | 10 | 25 | 10 | 18 | 39 | | 110 |
| **Richness (NE)** | - | **11** | **9** | **12** | **10** | **12** | **10** | **12** | **11** | | **14** |
| **TOTAL (NE)** | - | **86** | **90** | **117** | **187** | **202** | **139** | **292** | **332** | **1445** | |

**References**

1 George, D.R.; Croft, P.; Northing, P.; Wäckers, F.L. Perennial field margins with combined agronomical and ecological benefits. *IOBC/WPRS Bull.* **2010**, *56*, 45–48.

2 Wyss, E. The effects of artificial weed strips on diversity and abundance of the arthropod fauna in a Swiss experimental apple orchard. *Agric. Ecosyst. Environ*. **1996**, *60*, 47–59, doi:10.1016/S0167-8809(96)01060-2.

3 Larsson, M. Higher pollinator effectiveness by specialist than generalist flower-visitors of unspecialized Knautia arvensis (Dipsacaceae). *Oecologia* **2005**, *146*, 394–403, doi:10.1007/s00442-005-0217-y.

4 Carvell, C.; Meek, W.R.; Pywell, R.F.; Goulson, D.; Nowakowski, M. Comparing the efficacy of agri-environment schemes to enhance bumble bee abundance and diversity on arable field margins. *J. Appl. Ecol*. **2007**, *44*, 29–40, doi:10.1111/j.1365-2664.2006.01249.x.

5 Mullinix, K.; Isman, M.B.; Brunner, J.F. Key and Secondary Arthropod Pest Population Trends in Apple Cultivated over Four Seasons with No Insecticides and a Legume Cover. *J. Sustain. Agric*. **2010**, *34*, 584–594, doi:10.1080/10440046.2010.493363.

6 Hickman, J.M.; Wratten, S.D. Use of Phelia tanacetifolia Strips To Enhance Biological Control of Aphids by Overfly Larvae in Cereal Fields. *J. Econ. Entomol*. **1996**, *89*, 832–840.

7 Witjes, S.; Eltz, T. Hydrocarbon footprints as a record of bumblebee flower visitation. *J. Chem. Ecol.* **2009**, *35*, 1320–1325, doi:10.1007/s10886-009-9720-7.

8 Nakano, C.; Washitani, I. Variability and specialization of plant-pollinator systems in a northern maritime grassland. *Ecol. Res.* **2003**, *18*, 221–246, doi:10.1046/j.1440-1703.2003.00550.x.

9 Laverty, T.M. Costs to foraging bumble bees of switching plant-species. *Can. J. Zool.-Revue Can. Zool.* **1994**, *72*, 43–47.

10 Campbell, A.J.; Biesmeijer, J.C.; Varma, V.; Wäckers, F.L. Realising multiple ecosystem services based on the response of three beneficial insect groups to floral traits and trait diversity. *Basic Appl. Ecol.* **2012**, *13*, 363–370.

11 Colley, M.R.; Luna, J.M. Relative Attractiveness of Potential Beneficial Insectary Plants to Aphidophagous Hoverflies (Diptera: Syrphidae). *Environ. Entomol*. **2000**, *29*, 1054–1059, doi:10.1603/0046-225X-29.5.1054.

12 Begum, M.; Gurr, G.M.; Wratten, S.D.; Hedberg, P.R.; Nicol, H.I. Using selective food plants to maximize biological control of vineyard pests. *J. Appl. Ecol.* **2006**, *43*, 547–554, doi:10.1111/j.1365-2664.2006.01168.x.

13 Wäckers, F. L. Assessing the suitability of flowering herbs as parasitoid food sources: Flower attractiveness and nectar accessibility. Biol. Control 2004, 29, 307–314, doi:10.1016/j.biocontrol.2003.08.005.

14 Pontin, D.R.; Wade, M.R.; Kehrli, P.; Wratten, S.D. Attractiveness of single and multiple species flower patches to beneficial insects in agroecosystems. *Ann. Appl. Biol.* **2006**, *148*, 39–47, doi:10.1111/j.1744-7348.2005.00037.x.

15 Vattala, H.D.; Wratten, S.D.; Phillips, C.B.; Wäckers, F.L. The influence of flower morphology and nectar quality on the longevity of a parasitoid biological control agent. *Biol. Control* **2006**, *39*, 179–185, doi:10.1016/j.biocontrol.2006.06.003.

16 Lopez, R.; Merle Shepard, B. Arthropods associated with medicinal plants in coastal south carolina. *Insect Sci*. **2007**, *14*, 519–524, doi:10.1111/j.1744-7917.2007.00181.x.

17 Brown, M.W. Applying principles of community ecology to pest management in orchards. *Agric. Ecosyst. Environ.* **1999**, *73*, 103–106, doi:10.1016/S0167-8809(99)00018-3.

18 Géneau, C.E.; Wäckers, F.L.; Luka, H.; Daniel, C.; Balmer, O. Selective flowers to enhance biological control of cabbage pests by parasitoids. *Basic Appl. Ecol*. **2012**, *13*, 85–93, doi:10.1016/j.baae.2011.10.005.

19 Blake, R.J.; Westbury, D.B.; Woodcock, B.A.; Sutton, P.; Potts, S.G. Investigating the phytotoxicity of the graminicide fluazifop-P-butyl against native UK wildflower species. *Pest Manag. Sci*. **2012**, *68*, 412–421, doi:10.1002/ps.2282.

20 Rose, F. *The Wild Flower Key: How to Identify Wild Plants, Trees and Shrubs in Britain and Ireland, Revised Edition*.; Penguin Books: London, UK; **2006**.

© 2017 by the authors. Submitted for possible open access publication under the
terms and conditions of the Creative Commons Attribution (CC BY) license (http://creativecommons.org/licenses/by/4.0/).
